# Reconstruction of unfixable comminuted posterior wall acetabular fractures with autologous bone graft: A systematic review

**DOI:** 10.1016/j.jor.2024.10.034

**Published:** 2024-10-24

**Authors:** Riccardo Giai Via, Matteo Giachino, Ahmed Elzeiny, Alessandra Cipolla, Andrea Marino, Andrea D'Amelio, Francesco Bosco, Kristijan Zoccola, Alessandro Aprato, Alessandro Massè

**Affiliations:** a University of Turin, Centro Traumatologico Ortopedico (CTO), Department of Orthopaedic Surgery, Turin, Italy; b Department of Orthopaedics and Traumatology, Faculty of Medicine, Kafr El Sheikh University, Egypt; c Department of Precision Medicine in Medical, Surgical and Critical Care (Me.Pre.C.C.), University of Palermo, Palermo, Italy; d Department of Orthopaedics and Traumatology, G.F. Ingrassia Hospital Unit, ASP 6, Palermo, Italy; e Ortopedia e Traumatologia 2 – Ospedale San Giovanni Bosco, Azienda Sanitaria Locale Città di Torino, Turin, Italy; f University of Turin, Ospedale Infantile Regina Margherita, Department of Pediatric Orthopaedic Surgery, Turin, Italy

**Keywords:** Trauma, Acetabulum, Pelvic, Grafts, Fracture

## Abstract

**Background:**

Posterior wall acetabular fractures, often caused by high-energy trauma, are complex injuries that pose significant surgical challenges, especially when comminuted. Traditional fixation techniques have shown variable outcomes, with severe comminution sometimes rendering fragment fixation impossible. The aim of this study was to evaluate the clinical and radiological outcomes of autologous bone grafting for reconstructing severely comminuted unfixable posterior wall acetabular fractures.

**Materials and methods:**

A systematic review was conducted in accordance with the PRISMA guidelines. The search for clinical studies was carried out across four databases: Embase, PubMed, Medline, and Scopus. The included studies were evaluated using the Coleman Methodology Score. The present study protocol was registered in PROSPERO.

**Results:**

The study involved 71 patients, with an average age of 37.12 years. Autologous iliac crest grafts were predominantly used, with the Kocher-Langenbeck approach in all cases. Clinical outcomes, assessed by Merle d'Aubigne and Harris Hip Scores, showed 78.9 % of patients reporting excellent to good results. Radiological outcomes indicated 66 % with excellent results per Matta's score. The overall success rate ranged from 57 % to 100 %, with a 5 % conversion to total hip arthroplasty. Complications were reported in 7 % of cases, including nonunion and avascular necrosis.

**Conclusion:**

Autologous bone grafts for comminuted, non-fixable posterior wall acetabular fractures may be considered as a potential salvage option in young patients, potentially delaying the need for THA.

## 1 Introduction

Posterior wall acetabular fractures are typically due to high-energy trauma. According to Letournel and Judet's studies, these fractures are the most common type and account for about a quarter of all acetabular fractures. Only 30 % of posterior wall acetabular fractures involve a single large fragment; most are multi-fragmentary or have impaction areas.^1^

The most frequently employed approach involves anatomically reduction of joint fragments and stabilization, with or without the use of bone grafts (such as iliac crest autografts following the removal of comminuted fragments), to reinforce subchondral support. This technique was first introduced by Daum in 1993 through two case reports.^2^ Additionally, the use of a two-level reconstruction approach has become increasingly prevalent, involving subchondral mini screws to stabilize the elevated posterior marginal impaction, supplemented by further fixation using screws or plates. Thin, low-profile plates are frequently used as flexible or spring plates to stabilize fragmented bones. However, the superiority of these fixation methods is still a topic of debate.^3^ These techniques are highly technical, demanding a high level of surgical skill, an in-depth knowledge of fracture patterns, and access to a broad array of implants, all of which may contribute to the inconsistencies in fracture fixation outcomes.^4^

Comminuted posterior wall fractures have an exceptionally high risk of failure and poor outcomes, regardless of the reduction quality. Studies have shown that load-bearing acetabular roof involvement, marginal impaction, posterior hip dislocation, and femoral head injury are also risk factors for poor clinical outcomes.^5^

Some fractures have such severe comminution of the posterior wall that adequate fixation of the fragments is impossible. Arthroplasty may be proposed in these cases to elderly patients (over 65 years of age), mainly when associated with significant femoral head damage.^6^ It has been shown that 21–32 % of patients have poor results, with failure rates reaching up to 30 % within one year of posterior wall fracture fixation. The main treatment method in these cases is total hip arthroplasty (THA). However, these fractures often occur in young, active patients, who may face implant failure or numerous revisions later in life.^7^

Despite being amenable to anatomical reduction, comminuted fractures present a considerable risk of early failure or subsequent complications. Therefore, based on radiological planning and an intraoperative evaluation of the extent of comminution and the potential for fragment fixation, the possibility of reconstructing the comminuted fragments primarily using ipsilateral autologous bone graft can be considered^8^

The aim of this study was to evaluate the clinical and radiological outcomes of replacing severely comminuted and non-fixable posterior wall acetabular fractures by using bone grafts in young patients presenting with such fractures.

## 2 Materials and methods

This study adhered to the guidelines set forth by PRISMA.^9^ The literature review and study evaluation process were conducted independently by a team of two researchers (RGV and AE) to ensure accuracy. In case of ambiguities, a third author (MG) was consulted to resolve any uncertainties.

### 2.1 Inclusion and exclusion criteria

The selection criteria concentrated on research that involved patients with posterior wall fractures managed with bone grafts to repair the defect. The selected studies were required to be published in English, involve human participants, have publication dates between January 2004 and April 2024, and include a minimum mean follow-up period of 12 months. Articles prior to 2004 were excluded to ensure a more modern and up-to-date literature base for the systematic review, incorporating the most recent research. The analysis incorporated randomized controlled trials (RCTs) alongside prospective and retrospective studies with levels of evidence (LoE) ranging from 1 to 4.^10^ To ensure a higher quality of evidence in the review, studies focusing on biochemical or in vitro experiments, editorials, technical documents, preclinical studies, review papers were excluded.

### 2.2 Search strategy and study screening

**Fig. 1:**
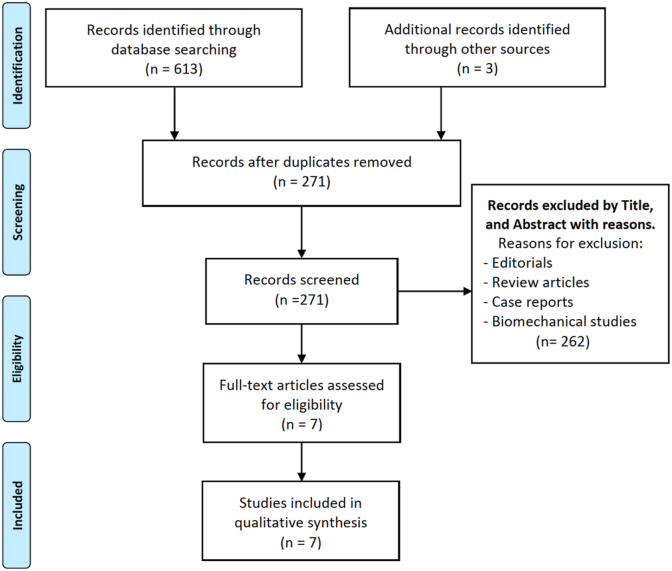
Prisma flow diagram.

A detailed literature search was conducted in May 2024 through five databases (PubMed, Scopus, Embase, Medline, and Cochrane), using the following MeSH terms: “posterior wall defect”, “posterior wall fracture”, “posterior wall”, “graft”, “bone graft”, “allograft”, “rib graft”, “acetabulum”, “pelvis”. After eliminating duplicates, 271 studies were initially identified. Following a review of the titles and abstracts, 264 studies were excluded, leaving 7 studies that met the eligibility criteria. A thorough evaluation of the full texts confirmed that these 7 clinical studies were suitable for qualitative analysis. The selected studies provided direct data on clinical and functional outcomes, radiological findings, associated injuries, postoperative rehabilitation programs, time to return to activity, as well as success and revision rates. The PRISMA flow diagram is illustrated in Fig. 1.

### 2.3 Methodological quality assessment

**Fig. 2:**
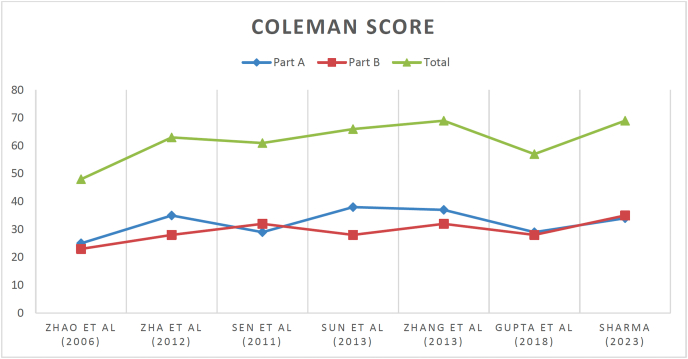
The Coleman Methodology Score (mCMS), modified by Ramponi et al..^10^

Each selected study was evaluated based on the levels of evidence (LoE), which range from 1 to 5.^9^ Retrospective studies were examined using the Coleman Methodology Score (mCMS)^11^^,^^12^ (Fig. 2). The assessment was conducted by two authors, with a third author being consulted to resolve any disagreements or uncertainties. All contributors were actively involved in the conceptualization and design of the study, as well as in data collection, manuscript drafting, and final revisions. Each author reviewed and gave their approval for the final manuscript. The study was registered with PROSPERO in May 2024.^13^

### 2.4 Data extraction

Two authors (RGV and AE) independently extracted and systematically documented data from the selected studies using Excel spreadsheets before merging their results. The collected information encompassed a wide range of details, including author names, publication dates, sample sizes, average patient age, and BMI. Other data points included the mechanism of injury, time elapsed before surgery, related injuries, average follow-up duration, patient positioning during surgery, the surgical techniques employed, and the post-operative rehabilitation protocols. Additionally, the data collection covered success rates, complication and revision rates, time required to resume activity, radiological outcomes based on Matta's score, and both pre- and post-operative subjective assessments using the Harris Hip Score (HHS), the Merle d'Aubigné and Postel score, and its modified version.

### 2.5 Statistical analysis

Statistical analysis was performed using R software (version 4.1.3, released in 2022, Vienna, Austria). Mean values were used to analyze continuous variables, with measures of variability such as standard deviation (SD) or range (minimum-maximum) also being reported. Categorical variables were analyzed by calculating absolute counts and their corresponding frequency distributions.

## 3 Results

The database search retrieved 616 articles. In the end, seven clinical studies met all inclusion criteria. They were eligible for critical analysis, quality assessment, and inclusion in the review on functional outcomes of patients undergoing comminuted posterior wall fracture reconstruction with autologous bone graft. All were published between 2006 and 2023 and were case series (Level IV) of evidence.^8^^,^^14^, ^15^, ^16^, ^17^, ^18^, ^19^ One study is a prospective case series,^15^ while the other six are retrospective.^8^^,^^14^^,^^15^^,^^17^, ^18^, ^19^

Of the 71 patients in this review, 31 were male and 11 female (data available from 6 studies).^8^^,^^14^, ^15^, ^16^^,^^18^^,^^19^ The mean patient age was 37.12 years, with a range from 8 to 58 years. Follow-up periods extended from 6 months to as long as 9 years in one long-term study. Only Sharma et al. specified the diseased side: right in 9 patients and left in 5. The time interval between injury and surgery was reported in five studies and ranged from 2 days to 11 months in neglected cases.^8^^,^^14^^,^^15^^,^^18^^,^^19^ The mechanism of injury was indicated in three studies, and most injuries were due to traffic accidents or falls from height. In addition, five studies reported associated injuries in addition to acetabular fractures of the posterior wall^8^^,^^14^^,^^15^^,^^18^^,^^19^ (Table 1).

**Table 1:** Demographic data of included studies and patients: N°: Number of patients; LoE: Level of Evidence;/: not reported; FU: Follow-up; FFH: falling from height; RTA: road traffic accident; #: fracture; MCA: motor car accident.

Authors (year)	Study design, (LoE)	N° of patients	Age years, Mean (Range Min-Max)	Male/Female, N°/N°	Mechanisms of injury, (N°)	FU years, Mean (Range Min-Max)	Time till surgery, Mean (Range Min-Max)	Associated injury, N°
Zhao et al. (2006)^13^	Retrospective (IV)	9	41.3 (28–54)	7/2	RTA (6) fall (3)	(6–9)	8 months (4–11)	5 (multiple #)
Zha et al. (2012)^14^	Retrospective (IV)	7	31 (8–53)	5/2	/	6.3 (0.7–20)	6.4 months (3–11)	/
Sen et al. (2011)^7^	Retrospective (IV)	8	36.6 (22–48)	7/1	/	3.34 (2–5)	5 days (2–14)	5 (femoral head #), medial malleolus #, patella #, forearm #, tibial plateau #, knee ligament injury)
Sun et al. (2013)^15^	Prospective (IV)	6	41.8 (16–62)	5/1	/	1.3 (0.5–2.2)	/	3 (femoral head #; tibia shaft #, fibular head #, 4th 5th metacarpal #, sciatic nerve injury)
Zhang et al. (2013)^16^	Retrospective (IV)	21	36.4 (18–58)	/	MCA, motorcycle crash and falls	6.3	/	/
Gupta et al. (2018)^17^	Retrospective (IV)	6	30.5 (18–49)	5/1	/	3.9 (3.6–4.8)	3 patients 1 week, 3 patients 2 weeks	3 (shaft femur #, ipsilateral bimalleolar #, sciatic nerve injury)
Sharma et al. (2023)^18^	Retrospective (IV)	14	42.2 (26–54)	10/4	RTA (12) FFH (2)	3.5 (2–7)	6 days (2–14)	5 (Blunt trauma chest, bone forearm #, head injury, abdominal injuries)

### 3.1 Indications

In all studies, the indication for posterior wall replacement with autologous bone graft was unresolvable and severely comminuted posterior wall acetabular fractures. Sharma et al. reported a mean number of fragments of 4.5, with an average fragment ratio of 1.6: 1.9: 2.4.^19^ Six studies used an autologous iliac crest graft for posterior wall replacement.^8^^,^^14^^,^^15^^,^^17^, ^18^, ^19^ However, Sun et al. used an autologous intertrochanteric ridge graft with gluteus medius muscle (Table 2).^16^

**Table 2:** Surgical technique, graft harvesting and postoperative therapy of patients in the included studies. N°: number of evaluation cases; KL: Kocher-Langenbeck; ASIS; anterior superior iliac spine, WB: weight bearing; ROM: range of motion; ATMFS: acetabular tridimensional memory alloy fixation system.

Authors (year)	Surgical technique	Graft harvesting and preparation	Postoperative rehabilitation
Zhao et al. (2006)^13^	KL approach Fixation with 2–3 lag screws + head fixation with k-wire	Iliac crest graft w harvested with periosteum which sculpted with a rongeur to conform defect, iliac fossa surface was placed toward the femoral head.	Postoperative shin traction and abduction position.
Zha et al. (2012)^14^	KL approach + head transfixation with k-wire Fixation with lag screws and plate	Full-thickness iliac crest, iliac crest side carved into a fish scale shape to improve graft healing, iliac fossa surface placed toward the femoral head.	Quadriceps exercises were encouraged. The hip spica cast taken off at 6 weeks, transfix K-wire was taken off at 3 months
Sen et al. (2011)^7^	KL approach (N°: 7) Ganz surgical dislocation (N°: 1) with pipkin # Fixation with lag screws and plate	Strut of iliac crest graft prepared in a step cut/oblique manner.	Traction lower limb for 3 weeks. Toe touch with crutches after 6 weeks. Complete WB after radiological union.
Sun et al. (2013)^15^	KL approach Fixation with lag screws and plate	Gluteus medius muscle-pedicled intertrochanteric crest graft.	Skin traction for first 2 weeks.
Zhang et al. (2013)^16^	KL approach + trochanteric flip osteotomy in some cases Fixation with ATMFS	Iliac crest graft after Ilium reaming with acetabular reamer 5-mm away from ASIS to be 3–4 mm thickness.	Mobilized without WB for 4 weeks. Partial WB, toe-touch with crutches allowed for 3 months. Gradual ROM 2nd day.
Gupta et al. (2018)^17^	KL approach + trochanteric flip osteotomy in all cases Fixation with lag screws and plate	Iliac crest strut graft prepared by nibbler.	Mobilized toe-touch with walker the next day.
Sharma et al. (2023)^18^	KL approach Fixation with lag screws and plate	Tricortical iliac crest graft is harvested as a single piece prepared using the Midas Rex® electrical system, smooth inner table towards the head.	Strict non-WB for 6 weeks followed by toe-touch partial WB on the affected side. Full-WB after 3 months.

### 3.2 Surgical technique

The Kocher-Langenbeck approach was used in all studies, with patients positioned in lateral decubitus facilitating graft harvesting.^8^^,^^14^, ^15^, ^16^, ^17^, ^18^, ^19^ Only Sen et al. used the surgical hip dislocation described by Ganz in a patient with an associated type I Pipkin fracture of the femoral head.^8^ The detailed description of the treatment received the maximum score of 10 points according to Coleman's score in two studies.^16^^,^^19^ Sharma et al. was the only study to mention the average operative time for the procedure and the amount of blood lost, with a time of 160 min (range: 125–190 min) and the associated blood loss, which was recorded at 410 ml (range: 320–830 ml).^19^

The method of bone graft fixation involved lag screws in addition to contoured plates in 6 studies.^8^^,^^14^, ^15^, ^16^^,^^18^^,^^19^ However, Zhang et al. used fixation with the three-dimensional shape-memory alloy acetabular fixation system (ATMFS),^17^ while Zhao et al. and Zha et al. used head prefixation with K-wires to ensure fixation.^14^^,^^15^ Table 2 shows the detailed surgical techniques and post-operative therapies.

### 3.3 Clinical and radiological assessment

Clinical outcomes were assessed using the Merle de'Aubigné score in six studies,^8^^,^^15^, ^16^, ^17^, ^18^, ^19^ while Zhao et al. utilized the Harris Hip Score (HHS).^14^ Of the 71 patients evaluated, 56 (78.9 %) achieved excellent or good results. Radiological outcomes, assessed using the Matta score in six studies, showed excellent to good results in 41 out of 62 patients (66 %) with follow-up times varying between 6 months and 20 years^8^^,^^15^, ^16^, ^17^, ^18^, ^19^ (Table 3).

**Table 3:** Summary of post operative outcomes, complications, success rate and revisions of the included studies. N°: number of evaluation cases; HHS: Harris hip score; HO: heterotrophic ossification; OA: osteoarthritis; AVN: avascular necrosis;/: not reported; Min: minimum; Max: maximum; preop: preoperative; postop: postoperative.

Authors (year)	Clinical results, N° (%)	Radiological results (Matta score), N° (%)	Time to return to activity (months), Mean (Range Min-Max)	Success rate, N°/N° (%)	Revision, N°	Complications, N°	THA conversion, N°
Zhao et al. (2006)^13^	**HHS preop,** Mean (Range Min-Max): 32.3 (23–56) **HHS postop,** Mean (Range Min-Max): 81 (59–93) Excellent in 3 patients, good in 4 patients, fair in 2 patients.	/	/	7/9 (77.8 %)	0	2: nonunion	0
Zha et al. (2012)^14^	**Merle de'Aubigne score:** Excellent for a pediatric patient, good for 3 adults' patients without post-traumatic OA and poor for 3 adults' patients with post-traumatic OA	Good in the pediatric patient, fair in 3 adults' patients without post-traumatic OA and poor in 3 adults' patients with post-traumatic OA.	/	4/7 (57 %)	0	0	3
Sen et al. (2011)^7^	**Merle de'Aubigne score:** Excellent in 2 (25 %) patients, very good in 2 (25 %) patients, good in 3 (37.5 %) patients, and fair in 1 (12.5 %) patient.	Excellent in 1 (12.5 %) patient, good in 4 (50 %) patients, fair in 3 (37.5 %) patients.	3.2	7/8 (87.5 %)	0	1: AVN	0
Sun et al. (2013)^15^	**Merle d’Aubigné score:** Excellent in 2 patients, and good in 4 patients.	Excellent in 2 patients, good in 4 patients.	/	6/6 (100 %)	0	1: HO (grade 1) 1: sciatic nerve injury	0
Zhang et al. (2013)^16^	**Merle D'Aubigné score:** Excellent in 7 patients, good in 10 patients, fair in 3 patients and poor in 2 patients.	Excellent in 10 patients, good in 8 patients, fair in 3 patients.	1.6 (1.2–2.1)	21/21 (100 %)	0	0	0
Gupta et al. (2018)^17^	**Merle d’Aubigné score:** Good in 4 (66.7 %) patients and poor in 2 (33.3 %) patients.	Good in 3 (50 %) patients, fair in 1 (16.7 %) patient, and poor in 2 (33.3 %) patients.	/	4/6 (66.7 %)	1: Excision arthroplasty	1: infection	1
Sharma et al. (2023)^18^	**Merle d’Aubigné score:** Excellent in 3 (21.4 %) patients, very good in 6 (42.9 %) patients, good in 2 (14.3 %) patients, and fair in 3 (21.43 %) patients.	Excellent in 2 patients (14.5 %), good in 6 patients (42.9 %), and fair in 6 patients (42.9 %).	3.4	14/14 (100 %)	0	1: HO (grade 2) 1: sciatic nerve injury	0

### 3.4 Success rates and complications

The success rates of the included studies ranged from 57 % to 100 %. 4 out of 71 patients (5 %) needed conversion to THA. Gupta et al. reported an excision arthroplasty in one patient.^4^ Time to return to activity was reported in 3 studies and ranged from 1.6 to 3.2 months.^8^^,^^17^^,^^19^ Some complications were reported in 5 studies.^8^^,^^14^^,^^16^^,^^18^^,^^19^ Zhao et al. reported nonunion in 2 patients,^14^ and Sen et al. reported avascular necrosis in one patient.^8^ Sun et al. and Sharma et al. reported one patient with heterotrophic ossification and one with sciatic nerve injury.^16^^,^^19^ Gupta et al. reported one patient with post operative infection^18^ (Table 3).

## 4 Discussion

Achieving anatomic reduction for acetabular fractures is essential, as demonstrated by Matta et al.,^20^ who found that the most important prognostic indicator is the quality of reduction: poor fracture reduction (>3 mm) is closely correlated with poor clinical outcomes, predisposing to femoral head wear, osteonecrosis, and early post-traumatic osteoarthritis. Another essential goal of surgical treatment is to reconstruct the anatomical joint surface, especially in the weight-bearing area, to restore normal stress distribution and physiological mechanics of load transmission through the hip. Both anatomical reduction and proper mechanical load distribution influence the incidence of post-traumatic arthritis.^21^

Fracture patterns like comminuted fractures of the posterior acetabular wall are particularly challenging for surgeons, especially in younger patients, for whom Letournel et al. reported only 35 % good clinical outcomes.^22^ One of the main issues with ORIF of the posterior wall is the blood supply damage caused by the detachment of the soft tissues, for fragments manipulation and reduction.^8^ Along with that in most of the cases the surrounding soft tissues are primarily damaged in the trauma, and the fragments are consequently already de-vascularized.^23^^,^^24^

Several techniques have been proposed to fix comminuted fractures of the posterior wall, such as the use of mini-screws and spring plates.^5^ Daum in 1993 and Sen et al., in 2011 described the use of autologous grafts, reporting satisfactory results (87.5 % clinical outcome) in a series of patients in which comminuted fragments were removed and replaced by an iliac crest graft fixed with plate and screws.^2^^,^^8^ The main advantages of this strategy include improved stability of the fixation in the early post-operative period, elimination of potential loose osteochondral fragments, and restoration of a new proper posterior wall continuity. Additionally, in cases of post-traumatic osteoarthritis, the graft may integrate, providing adequate bone stock for a prosthetic implant. However, the technique has several drawbacks: the graft is not vascularized and may be prone to bone resorption, the articular cartilage is not restored, and the femoral head will articulate directly with the bone surface. There is also potential morbidity at the graft harvest site.

Our review, including 71 patients, found a success rate of 84 % (range: 57 %–100 %). Clinical satisfaction was excellent or good in 78.9 % of patients. Overall, only 4 patients (5 %) needed conversion to THA with a mean follow-up of 3.6 years (6 months - 9 years).

All patients were treated with a Kocher-Langenbeck approach in the lateral position to facilitate graft harvesting. In 65 patients (91.5 %), an iliac crest graft was used because, although it cannot replace the cartilaginous surface, the biological characteristics of cancellous iliac bone are the most similar to posterior wall reconstruction and offer the advantage of easy harvesting.^15^ In 6 patients (8.5 %), an intertrochanteric ridge graft with gluteus medius muscle pedicle was used because, according to the author, it could be better shaped to fit the posterior wall defect and provide better structural support.^16^

There is no statistical evidence of which graft is better, as there is no consensus on which method of graft fixation is best. In 41 patients (57.7 %), the graft was fixed with lag screws and contoured plates. In 9 patients (12.7 %), only lag screws were used. In 21 patients (29.6 %), an acetabular tridimensional memory alloy fixation system (ATMFS) was used in addition to lag screws. Although there is no statistical evidence, a consensus asserted that lag screws combined with a reconstruction plate achieved a less stressful fixation compared to spring plates and, at the same time, allowed a more stable fixation compared to lag screws alone without a buttress plate.^19^^,^^25^ Regardless of the method, rigid internal fixation is essential for graft healing in the early post-operative period.^15^

A key point that deserves attention concerns the anatomical reductions reported by the authors. Although these reductions may appear evident on radiographs, it is unlikely that they are real, given that a graft has been used. Radiographic images may give the impression of a reduction on X-Rays, but a more detailed examination by CT or MRI would reveal the presence of large gaps, calling the authors' conclusions into question.^29^

In other words, one must be cautious in accepting claims of anatomical reduction. The use of a graft could make these posterior wall reductions seem real, but only a more in-depth analysis, such as a CT scan or MRI, could reveal the real situation, showing the presence of large gaps and proving that the observations made are not entirely accurate.^29^

Even with proper reduction, a significant number of patients with posterior wall comminuted fractures are at high risk of post-traumatic OA. In any case of a ‘joint at risk,' such as in elderly patients, in existing hip arthritis, in established post-traumatic arthritis or in severe cartilage injury, an acute THA must be at least considered.^26^ Despite the challenges in performing 10.13039/100026140THA in acetabular fracture context, the modern literature supports the acute ‘fix and replace' approach.^27^^,^^28^ Whether acute or delayed, THA is a safe option, providing good clinical outcomes, quality of life and lower risk of reoperation rates in patients with complex acetabular fractures.^29^

Reconstructing comminuted posterior wall acetabular fractures using the autologous bone graft technique should not be underestimated. It serves as a salvage option for younger patients when ORIF alone is likely to yield suboptimal results. However, this study has several limitations. First, the analysis includes only a small number of low-quality studies, with most being retrospective case series. Second, the studies demonstrate several potential biases related to data collection methods, participant selection, and the lack of blinding in outcome assessments, all of which may affect the reliability and validity of the results. Third, there was a wide range of follow-up periods reported, from 0.5 to 20 years, and more consistent and standardized clinical and radiological follow-ups could enhance the validity of the data. Fourth, surgeries were conducted by different surgeons, introducing potential bias due to differences in surgical techniques. Lastly, the rehabilitation protocols varied, and the lack of a standardized approach could affect post-operative outcomes. Future high-quality studies are needed to reinforce the findings of this review.

## 5 Conclusions

This study shows that the use of autologous bone grafts for comminuted, non-fixable acetabular fractures of the posterior wall can be considered as a potential salvage option in young patients, potentially delaying the need for THA. However, the success rate varies, with only 78.9 % of patients having excellent or good results and a fair proportion still requiring THA**.**

## CRediT authorship contribution statement

**Riccardo Giai Via:** Conceptualization, Writing – original draft. **Matteo Giachino:** Writing – original draft. **Ahmed Elzeiny:** Writing – original draft, Methodology. **Alessandra Cipolla:** Writing – original draft. **Andrea Marino:** Investigation, Visualization. **Andrea D'Amelio:** Visualization. **Francesco Bosco:** Visualization, Supervision. **Kristijan Zoccola:** Supervision. **Alessandro Aprato:** Visualization, Supervision. **Alessandro Massè:** Supervision.

## Level of evidence

IV.

## Ethical statement

The study was conducted following the ethical standards of the Declaration of Helsinki (1964).

## Guardian/Patient's consent

Not Applicable.

## Competing of interest

All authors have no conflicts of interest.

## Funding Statement

This research did not involve any specific grants from commercial, public, or non-profit sector funding agencies.

## Declaration of competing interest

On behalf of all authors, the corresponding author states that there is no conflict of interest.
